# β-Hydroxybutyrate attenuates glycation-induced structural destabilization and amyloidogenic aggregation in human serum albumin

**DOI:** 10.1016/j.bbrep.2026.102487

**Published:** 2026-02-09

**Authors:** Hojjat Mohammadnia, Mousa Bohlooli, Mansour Ghaffari-Moghaddam, Mostafa Khajeh, Fereshteh Taghavi

**Affiliations:** a Department of Chemistry, University of Zabol, Zabol, Iran; b Department of Cell and Molecular Sciences, Kharazmi University, Tehran, Iran; c Faculty of Biological Science, Tarbiat Modares University, Tehran, Iran

**Keywords:** Human serum albumin, β-Hydroxybutyrate, Advanced glycation end products, Structural stabilization, Amyloidogenic pathways, Atomic force microscopy, Circular dichroism spectroscopy, Neurodegeneration

## Abstract

Protein aggregation and amyloid fibril formation are key events in neurodegenerative disorders such as Alzheimer's disease, and are worsened by non-enzymatic glycation, which destabilizes protein structure. Glycation by reducing sugars like glucose produces advanced glycation end products (AGEs), promoting misfolding and aggregation. β-Hydroxybutyrate (BHB), a ketone body with anti-glycation and neuroprotective properties, may counteract these effects. This study examined structural, aggregation, and fibrillar changes of human serum albumin (HSA) after prolonged glucose-induced glycation, and evaluated the modulatory role of BHB via a multi-technique approach. HSA was incubated for 120 days under four conditions: control, BHB alone, glucose alone, and glucose + BHB. Analyses included atomic force microscopy (AFM), circular dichroism (CD) spectroscopy, ANS fluorescence, Thioflavin T (ThT) fluorescence, and Congo Red assays. AGE formation was quantified to link biochemical modifications with aggregation patterns. Glucose treatment markedly increased AGE levels. AFM revealed extensive aggregation and high surface coverage in the glucose group, partially reduced by BHB. CD spectroscopy showed α-helix loss and β-sheet enrichment with glycation, while BHB preserved structure. ANS fluorescence indicated glucose-enhanced hydrophobic exposure, reduced by BHB. ThT and Congo Red assays confirmed less amyloid fibril formation in glucose + BHB samples versus glucose alone. These results suggest that glucose induces marked glycation, structural disruption, and amyloidogenic aggregation in HSA, whereas BHB provides partial protection, likely through structural stabilization and aggregation pathway modulation. BHB may offer therapeutic promise for limiting amyloid-related neurodegenerative diseases.

## Highlights

• Glucose markedly increased AGE formation and amyloidogenic aggregation in HSA.
• Glucose-induced glycation of HSA promotes α-helix loss and β-sheet enrichment.
• Co incubation with BHB significantly reduced glucose induced aggregation.
• BHB preserved secondary structure and reduced surface hydrophobicity.
• BHB shows therapeutic potential for neurodegenerative disease prevention.

## Graphical abstract

**Figure:**
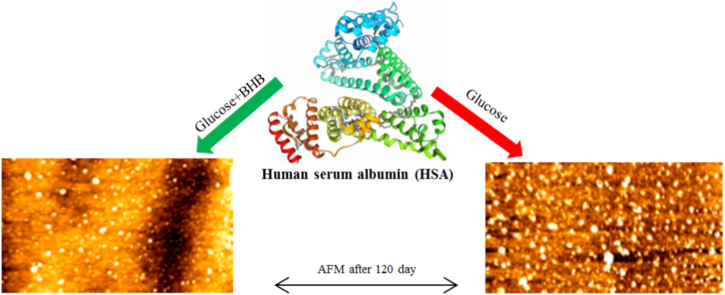

## 1 Introduction

Protein aggregation and amyloid fibril formation are fundamental pathological processes implicated in diverse human diseases, including neurodegenerative disorders, diabetes, cardiovascular pathologies, and metabolic syndromes [1,2]. In these conditions, proteins that predominantly adopt native α-helical conformations undergo structural destabilization, leading to the assembly of insoluble β-sheet-rich aggregates. Such aberrant assemblies disrupt cellular homeostasis, elicit immune responses, and ultimately cause cell death, as exemplified in Alzheimer's and Parkinson's diseases [3,4]. Elucidating the molecular mechanisms that drive aggregation, particularly amyloidogenesis, is crucial for understanding disease pathogenesis and designing targeted therapeutic strategies.

Human serum albumin (HSA), the most abundant plasma protein, maintains colloid osmotic pressure, transports fatty acids and pharmaceuticals, and modulates oxidative stress [5,6]. Its well-characterized structure and remarkable refolding capacity render it an ideal model for probing biophysical processes, including glycation and amyloid aggregation [7]. Importantly, glycation-induced amyloidogenic behavior of HSA is directly relevant to diabetic complications, including diabetic amyloidosis and vascular pathologies. Glycation promotes β-sheet enrichment and amyloid-like aggregation in HSA [8], processes that contribute to protein deposition and chronic inflammation in diabetes. Furthermore, advanced glycation end-products accelerate amyloid deposit formation in metabolic tissues [9], highlighting the pathological relevance of glycation-driven structural destabilization. Experimental evidence indicates that HSA is highly susceptible to conformational alteration and aggregation under deleterious conditions—such as hyperglycaemia and oxidative stress—highlighting its relevance for studying protein misfolding disorders and evaluating candidate protective agents [10].

Among the biochemical triggers of protein aggregation, non-enzymatic glycation (Maillard reaction) is of particular importance [9,11]. In this spontaneous reaction, reducing sugars bind to protein amino groups, giving rise to advanced glycation end products (AGEs) that profoundly alter charge distribution, conformational stability, and biological function [12,13]. Recent investigations have shown that prolonged glycation of HSA not only induces marked α-helix loss and β-sheet enrichment but also alters domain flexibility, hydration patterns, and ligand-binding capacity, thereby impairing its physiological functions [14,15]. These structural and functional changes are accompanied by enhanced surface hydrophobicity and amyloid-like cross-β aggregation, underscoring the relevance of glycated HSA in chronic hyperglycaemia.

Glycation reduces solubility, destabilizes native folding, and lowers the thermodynamic barrier to β-sheet formation, thereby accelerating amyloid fibril assembly [16,17]. Elevated levels of glycated proteins—including HSA—are reported in metabolic disorders and ageing, suggesting their potential as biomarkers for amyloid-associated complications [18]. Moreover, AGE accumulation can activate inflammatory cascades and oxidative stress pathways, amplifying disease progression.

A major focus in contemporary biophysical research is the identification of compounds capable of preventing AGE formation or mitigating their downstream structural and functional consequences. β-Hydroxybutyrate (BHB), a physiologically relevant ketone body, has emerged as a promising modulator with potential to counteract glycation-induced structural destabilization and aggregation [[19], [20], [21]]. Previous studies—including our own [22]—have shown that BHB inhibits HSA glycation, reducing AGE generation and associated conformational impairments. The protective effects of BHB may involve: (i) reducing the formation of reactive glycation intermediates, and (ii) antioxidant activity that attenuates oxidative stress accompanying AGE formation [23,24]. These mechanisms collectively may limit glycation-induced protein destabilization. However, whether these protective properties extend beyond AGE suppression to the modulation of amyloidogenic pathways in pre-glycated HSA remains unknown.

In the present study, we address this gap using an integrated methodological framework combining fluorescence spectroscopy, circular dichroism, Congo Red binding, and high-resolution atomic force microscopy (AFM). Critically, we demonstrate for the first time that BHB attenuates amyloidogenic aggregation in already-glycated HSA, extending beyond previous work focused solely on glycation inhibition. This approach enabled us to assess whether BHB can stabilize the native conformation of pre-glycated HSA and mitigate fibril assembly. Our findings provide new mechanistic insights into the interplay between glycation and aggregation, reinforcing the therapeutic promise of BHB in combating protein misfolding pathologies and informing strategies for the prevention and treatment of amyloid-related diseases.

## 2 Materials and methods

### 2.1 Chemicals and reagents

Human serum albumin (HSA, 96%, essentially free of fatty acids, Sigma-Aldrich, catalog no. A9511)**,** Thioflavin T (ThT, Sigma-Aldrich, catalog no. T3516)**,** Congo Red (CR, Sigma-Aldrich, catalog no. C6277)**,** and β-hydroxybutyric acid (BHB, Sigma-Aldrich, catalog no. 54965) were obtained from Sigma-Aldrich Company. 2,4,6-Trinitrobenzenesulfonic acid (TNBSA, 0.01%, Fluka, catalog no. 92822)**,** β-d-glucose (Fluka, catalog no. G7021)**,** and 1-anilinonaphthalene-8-sulfonate (ANS, Sigma-Aldrich, catalog no. A1028) were also obtained. Bicinchoninic acid (BCA, Sigma-Aldrich, catalog no. B9643) and bovine serum albumin (BSA, Sigma-Aldrich, catalog no. A2153) were used for protein quantification. EDTA (Sigma-Aldrich, catalog no. E9884) and sodium azide (Sigma-Aldrich, catalog no. S2002) were included in buffer preparations. All samples were prepared in 50 mM sodium phosphate buffer at pH 7.4. For each experimental procedure, human serum albumin (HSA) was used at a final concentration of 40 mg/mL—equivalent to its physiological level in human blood—and was freshly prepared prior to the initiation of the experiments [25]. All chemicals used in the study were of analytical grade and were employed as received, without additional purification.

### 2.2 The AGE-HSA preparation process

HSA was dissolved in a buffer comprising 50 mM sodium phosphate (pH 7.4), 1 mM EDTA, and 0.1 mM sodium azide to obtain a final concentration of 40 mg/mL. The glycation process started with the addition of 16.5 mM β-d-glucose, with or without 21.87 mM BHB. The concentration of BHB was chosen according to the values observed in fasting and patients with diabetes [26]. During 120 days, HSA was incubated with all reagents at 37 °C, pH 7.4 and in an atmosphere of darkness to mimic the physiological state of the cell. Human plasma's natural HSA environment was replicated by a dark environment. Following incubation periods of 35 and 120 days, all samples (HSA-control, HSA + BHB, HSA + Glc, and HSA + Glc + BHB) were subjected to dialysis for 48 h at 4 °C against 50 mM sodium phosphate buffer (pH 7.4) to remove unbound sugars and low-molecular-weight reaction products, thereby halting further glycation and equilibrating the samples in physiological buffer conditions, as described previously [17,18]. The dialyzed samples were subsequently stored at −30 °C until further analysis [25]. To measure the protein concentrations in the samples, bicinchoninic acid (BCA) was applied as an assay, while bovine serum albumin (BSA) utilized as a standard curve. The results were calculated as an average after each incubation was performed three times.

In the overall experimental design, most biophysical and biochemical characterizations (including AGE fluorescence, secondary structure, and surface hydrophobicity analyses) were conducted on samples incubated for 35 days, a time point previously shown to be optimal for detecting early glycation-induced structural changes [12]. In contrast, assays specifically aimed at assessing amyloid fibril formation (ThT fluorescence, Congo Red binding, and AFM imaging) were performed on samples incubated for 120 days to capture advanced aggregation states [18].

### 2.3 Quantification of protein glycation

#### 2.3.1 Determination of free lysine residues by TNBSA assay

The level of free amino groups in all HSA samples was quantified using the 2,4,6-trinitrobenzenesulfonic acid (TNBSA) assay, as previously described [27]. Briefly, protein samples were dissolved in 0.1 M sodium bicarbonate buffer (pH 8.5) at a final concentration of 0.2 mg/mL. To each 0.5 mL of protein solution, 0.25 mL of 0.01% (w/v) TNBSA was added and mixed thoroughly. The mixtures were then incubated at 37 °C for 2 h to allow for reaction. After incubation, 0.25 mL of 10% (w/v) SDS and 0.125 mL of 1 N HCl were sequentially added to each tube. The absorbance was measured at 335 nm against a blank (prepared identically but without albumin).

The degree of glycation (τ) was calculated using the following equation:

(1) $$\tau =\frac{\left({OD}_{control}-{OD}_{modified}\right)\times 58}{{OD}_{control}}$$

Where ${OD}_{control}$ and ${OD}_{modified}$ refer to the absorbances of the unmodified and modified (glycated in the present and absent of BHB) HSA samples, respectively.

#### 2.3.2 AGEs fluorescence measurement

The formation of advanced glycation end-products (AGEs) in protein samples was assessed by fluorescence spectroscopy. HSA samples were prepared at a concentration of 1.5 mg/mL in 50 mM sodium phosphate buffer (pH 7.4). Based on previous observations indicating that the fluorescence intensity of AGEs reaches its optimal detectability at approximately 35 days of incubation [12,28], this assay was performed on samples incubated for 35 days. Fluorescence spectra were acquired using a Cary Eclipse fluorescence spectrophotometer (Agilent Technologies) with excitation at 335 nm and emission recorded over the range of 300–600 nm. The excitation and emission slit widths were set to 5 nm and 10 nm, respectively. All measurements were conducted at room temperature, and each experiment was performed in triplicate.

### 2.4 Structural and conformational analysis

#### 2.4.1 Secondary structure analysis (CD spectroscopy)

An analytical spectropolarimeter (JASCO J-810 CD) with a 1-mm path length at 25 °C was used to measure circular dichroism (CD) in the samples. The samples were prepared in a 50 mM sodium phosphate buffer (pH 7.4) at 0.5 mg/mL concentration [29]. The obtained CD spectra were then converted to the mean residue ellipticity, ${\left[\theta \right]}_{\lambda }$, $\left(\deg.{\text{cm}}^{2}{.\text{mol}}^{-1}\right)$ at a specific wavelength λ (nm)using the following equation:

(2) $${\left[\theta \right]}_{\lambda }=\frac{\theta_{\lambda }M_{0}}{10.c.l}$$

In this equation, $\theta_{\lambda }$ represents the ellipticity (in millidegrees) at wavelength λ, $M_{0}$ is the average weight of residue, c refers to the concentration of protein (mg/mL), and l is the length of path length (cm). Based on CDNN CD Spectra Deconvolution Software (Version 2.1), the percentage of secondary structure was calculated [30].

#### 2.4.2 ANS fluorescence analysis

The surface hydrophobicity of protein samples was assessed using 1-anilinonaphthalene-8-sulfonate (ANS) as a fluorescent probe [25]. Protein samples were prepared at a final concentration of 0.2 mg/mL in 50 mM sodium phosphate buffer (pH 7.4). For each assay, 5 μL of a 2.5 mM ANS solution in the same buffer was added to the protein solution. The mixtures were incubated for 30 min at room temperature in the dark to promote binding. Fluorescence emission spectra were subsequently recorded from 300 to 600 nm with an excitation wavelength of 385 nm, using a Cary Eclipse spectrofluorometer (Agilent Technologies, USA). The fluorescence intensity at the emission maximum (typically 470–480 nm) was used to evaluate changes in the protein's surface hydrophobicity. This measurement was performed on protein samples that had been incubated for 35 days, a period previously reported to be optimal for detecting glycation-induced alterations in surface hydrophobicity [31,32]. All measurements were performed in triplicate, and control samples containing buffer and ANS without protein were used as blanks for baseline correction.

### 2.5 Amyloidogenic aggregation assessment

#### 2.5.1 Thioflavin T fluorescence assay

Amyloid fibril formation was assessed using the Thioflavin T (ThT) fluorescence assay, which exploits the enhanced fluorescence of ThT upon binding to the β-sheet-rich structures characteristic of amyloid fibrils [33]. After a 120-day incubation period, 30 μL of each sample (control and treated HSA, 1 mg/mL) was mixed with 100 μL of ThT solution (prepared in 100 mM sodium phosphate buffer, pH 7.4). The mixtures were incubated briefly at room temperature, and fluorescence emission spectra were recorded using an H4 spectrophotometer (BioTek, USA) with an excitation wavelength of 450 nm and emission measurement at 490 nm. Buffer blanks (sodium phosphate buffer without protein) were used to correct for background fluorescence from ThT.

#### 2.5.2 Congo red binding assay

At each designated time point, at each designated time point, 24 μL of the protein sample (prepared at 1 mg/mL) was mixed with 176 μL of a 20 μM Congo Red (CR) solution, prepared in 5 mM sodium phosphate buffer containing 150 mM NaCl (pH 7.4)**,** resulting in a final HSA concentration of approximately 0.12 mg/mL in the assay mixture. The use of 5 mM sodium phosphate buffer, rather than the 50 mM concentration used in other assays, was intentional: this lower ionic strength optimizes Congo Red-amyloid binding by minimizing competitive electrostatic interactions, while the 150 mM NaCl maintains physiological ionic strength [34]. The mixture was incubated at room temperature for 15 min to allow for dye-protein interaction. Absorbance spectra were then recorded from 400 nm to 700 nm using a UV-Vis spectrophotometer (UV-VIS-NIR; Cary UV-100, Varian, Australia) equipped with a 1 cm path length quartz cuvette [34]. Appropriate blanks containing buffer and CR without protein were used to correct for background absorbance.

#### 2.5.3 Atomic force microscopy (AFM) analysis

The morphology of HSA fibrils and aggregates formed under different SDS concentrations was studied using atomic force microscopy (AFM). Measurements were performed in non-contact mode, with the loop filter set at 2 Hz and the applied force ranging from 5.5 to 22.5 N/m. For sample preparation, 10 μL of each incubated protein solution (diluted to a final concentration of 0.002 mg/mL) was carefully deposited onto a freshly cleaved mica surface that had been thoroughly cleaned with deionized water and ethanol. The mica substrates were gently dried under a stream of nitrogen gas and stored at room temperature until analysis. AFM imaging was carried out using an Autoprobe CP Research system (Veeco, USA) equipped with a conical silicon tip (resonance frequency: 190–325 kHz). Scanning was performed over a 1 μm × 1 μm area. All images were collected and analyzed according to the manufacturer's protocols and reported as representative of at least three independent experiments [35].

### 2.6 Statistical analysis

All experiments were performed in triplicate (n = 3 independent measurements per condition), and results are expressed as mean ± standard deviation (SD). For quantitative analyses, raw data were baseline-corrected relative to the HSA control group, which was defined as the reference state in each experimental assay. Where applicable, data were normalized to the HSA-control condition prior to statistical comparison. Statistical significance among multiple experimental groups was evaluated using one-way analysis of variance (ANOVA), followed by Tukey's post hoc test for multiple comparisons. A significance threshold of p < 0.05 was applied throughout. Data distributions were inspected for consistency and comparable variance across groups, supporting the application of parametric statistical testing. Statistical analyses were performed using GraphPad Prism version 8.4.3 (GraphPad Software, San Diego, CA, USA). For circular dichroism (CD) spectroscopy, statistical comparisons were conducted between HSA control and each treatment group (HSA + BHB, HSA + Glc, HSA + Glc + BHB) for individual secondary-structure components (α-helix, antiparallel β-sheet, parallel β-sheet, β-turn, and random coil). For ThT fluorescence, Congo Red binding, and ANS fluorescence assays, fluorescence intensities or absorbance values were compared across all experimental groups using the same statistical framework.

## 3 Results

### 3.1 Quantification of protein glycation

#### 3.1.1 Lysine modification analysis

**Fig. 1:**
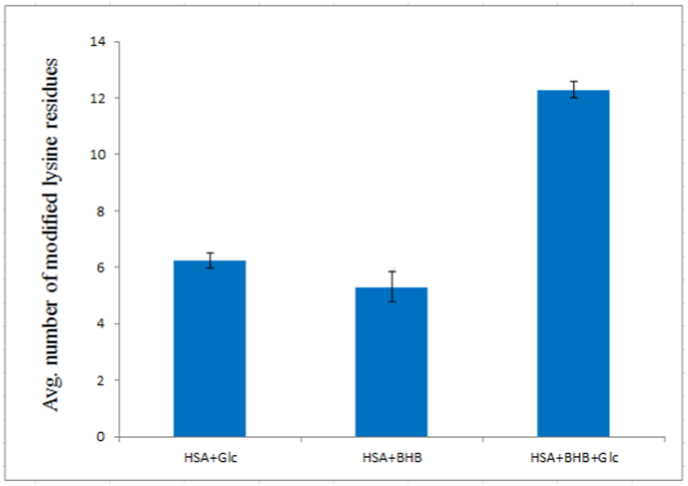
Average number of modified lysine residues in HSA + Glc, HSA + BHB and HSA + Glc + BHB after 35 days of incubation in 50 mM sodium phosphate buffer (pH 7.4), containing 1 mM EDTA and 0.1 mM sodium azide, at 37 °C. Values were calculated relative to HSA-control, which was used as the reference baseline for the TNBSA assay (modified lysine = 0). Data are presented as mean ± SD (n = 3). Statistical analysis was performed using one-way ANOVA followed by Tukey's post hoc test. Asterisks indicate significant differences compared to the reference control (p < 0.05). Statistical procedures are detailed in Section 2.6.

Quantitative assessment of lysine modification (Fig. 1) revealed covalent adduct formation in all three treatment groups. In the glucose-only samples, approximately six lysine residues per mole of HSA were modified. In the BHB-only group, modification involved around five residues per mole of protein. The combined glucose + BHB treatment yielded the highest modification level, with ∼12 lysine residues modified per mole of albumin.

#### 3.1.2 AGE-specific fluorescence

**Fig. 2:**
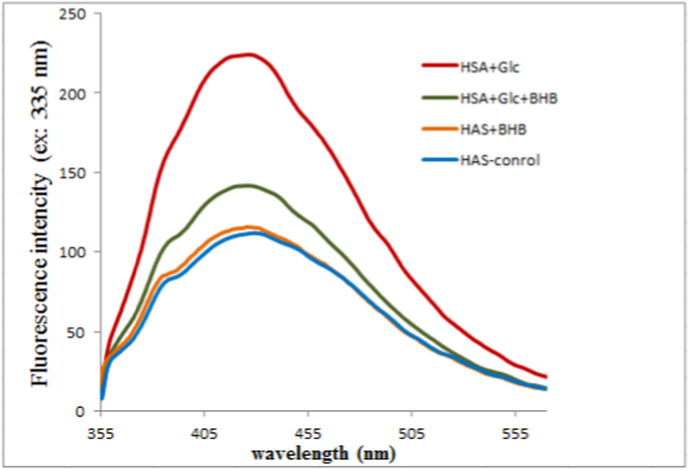
AGE-specific fluorescence emission spectra of HSA-control, HSA + BHB, HSA + Glc + BHB and HSA + Glc at an excitation wavelength of 335 nm after 35 days of incubation at 37 °C in 50 mM sodium phosphate buffer (pH 7.4). Data represent mean fluorescence intensity from three independent experiments (n = 3). Statistical comparisons between groups were performed at the emission maximum wavelength using one-way ANOVA followed by Tukey's post hoc test. Significant differences were considered at p < 0.05. Statistical procedures are detailed in Section 2.6.

Fluorescence spectra of control HSA, HSA + BHB, HSA + Glc, and HSA + Glc + BHB after 35 days of incubation at 37 °C are shown in Fig. 2. The HSA + Glc + BHB group exhibited lower fluorescence intensity across the emission range compared with the HSA + Glc group, indicating reduced accumulation of glycation-associated fluorescent adducts under co-treatment conditions.

### 3.2 Structural and conformational analysis

#### 3.2.1 Secondary structure analysis

**Fig. 3:**
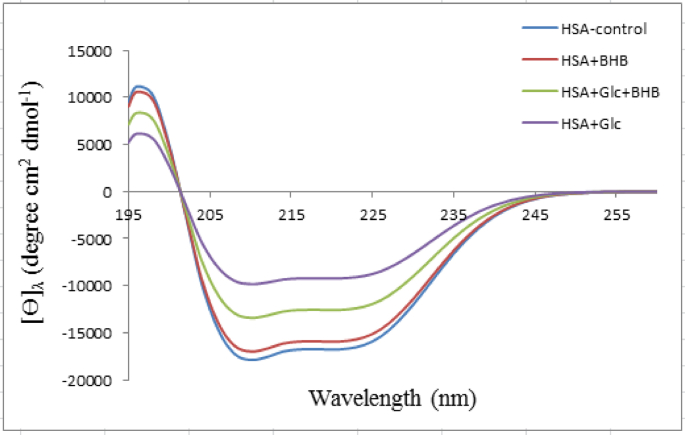
Far-UV circular dichroism spectra of HSA-control, HSA + BHB, HSA + Glc + BHB and HSA + Glc after 35 days of incubation at 37 °C in 50 mM sodium phosphate buffer (pH 7.4), 1 mM EDTA and 0.1 mM sodium azide. Data are presented as mean ± SD (n = 3). Statistical comparisons were made using one-way ANOVA followed by Tukey's post hoc test for each structural component. Significant differences (p < 0.05) were observed for specific secondary-structure components, as quantified in Table 1.

Far-UV circular dichroism (CD) spectroscopy was used to assess the secondary structure of HSA under four experimental conditions—HSA control, HSA + Glc, HSA + BHB, and HSA + Glc + BHB—after 35 days of incubation at 37 °C (Fig. 3). Spectral deconvolution, performed using the manufacturer's analysis software, provided the relative proportions of α-helix, β-sheet, β-turn, and random coil (Table 1).

**Table 1:** Percentage secondary-structure content of HSA-control, HSA + BHB, HSA + Glc + BHB, and HSA + Glc in 50 mM sodium phosphate buffer (pH 7.4) containing 1 mM EDTA and 0.1 mM sodium azide, after incubation at 37 °C for 35 days. Data are presented as mean ± SD (n = 3). Statistical analysis was performed using one-way ANOVA followed by Tukey's post hoc test. Significant differences (p < 0.05) were observed between the HSA + Glc group and control for α-helix and β-sheet components.

Sample	α−Helix	Antiparallel	Parallel	β−Turn	Random-coil
**HSA-control**	55.1% ± 0.4	6.3% ± 0.6	7.8% ± 0.3	15.1% ± 0.3	15.7% ± 0.7
**HSA + BHB**	53.4% ± 0.7	6.6% ± 0.5	8.2% ± 0.4	15.7% ± 0.4	16.1% ± 0.4
**HSA + Glc + BHB**	44.1% ± 0.3	9.3% ± 0.6	11.4% ± 0.4	17.1% ± 0.3	18.1% ± 0.5
**HSA + Glc**	33.6% ± 0.6	13.4% ± 0.5	14.5% ± 0.3	18.3% ± 0.4	20.2% ± 0.6

In the control HSA group, α-helix content was 55.1%, with total β-sheet at 14.1%. Glycation with glucose (HSA + Glc) reduced α-helix to 33.6% and increased total β-sheet to 27.9%, the highest value observed among all groups. The HSA + BHB group showed α-helix at 53.4% and β-sheet at 14.6%, indicating minimal change from the native fold. In the co-incubated HSA + Glc + BHB group, α-helix content was 44.8% and β-sheet was 20.7%, representing partial recovery of helical content and a reduction in β-sheet relative to glucose alone.

#### 3.2.2 ANS binding assay

**Fig. 4:**
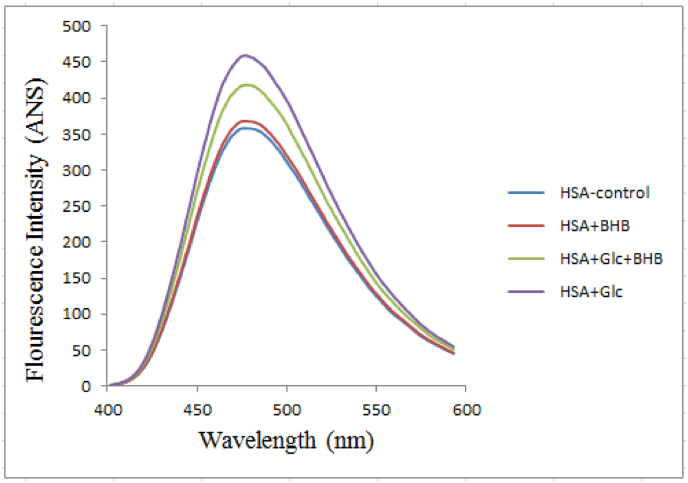
ANS fluorescence intensity spectra of HSA-control, HSA + BHB, HSA + Glc + BHB and HSA + Glc in 50 mM sodium phosphate buffer (pH 7.4), 1 mM EDTA and 0.1 mM sodium azide, incubated at 37 °C for 35 days. Data represent mean fluorescence intensity from three independent experiments (n = 3). Statistical comparisons between groups were performed at the emission maximum wavelength, selected a priori as the representative spectral feature.

Fluorescence emission spectra of HSA control, HSA + Glc, HSA + BHB, and HSA + Glc + BHB after 35 days of incubation at 37 °C are shown in Fig. 4. Samples were excited at an appropriate wavelength for ANS, and emission was recorded over the specified spectral range. The control HSA group exhibited the lowest fluorescence intensity, whereas HSA + Glc showed the highest. The HSA + Glc + BHB group displayed a reduction in fluorescence intensity relative to HSA + Glc alone.

### 3.3 Amyloidogenic aggregation assessment

#### 3.3.1 Amyloid fibril quantification (ThT fluorescence assay)

**Fig. 5:**
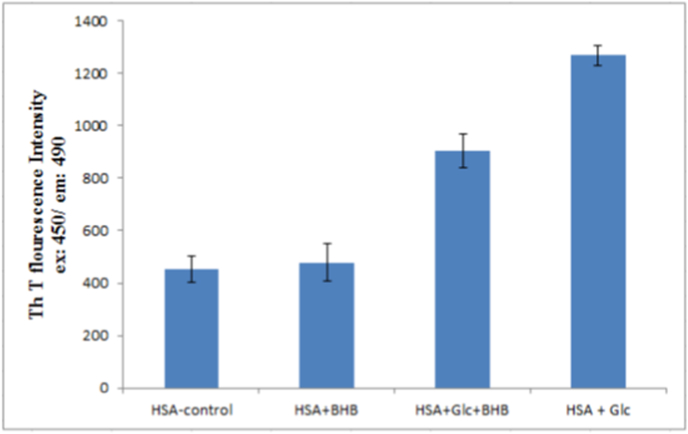
ThT fluorescence intensity reflecting amyloid fibril formation in HSA-control, HSA + BHB, HSA + Glc + BHB and HSA + Glc in 50 mM sodium phosphate buffer (pH 7.4), 1 mM EDTA and 0.1 mM sodium azide, incubated at 37 °C for 120 days. Data are presented as mean ± SD (n = 3). Statistical analysis was performed using one-way ANOVA followed by Tukey's post hoc test. Significant differences (p < 0.05) were observed between the HSA + Glc group and the other experimental conditions.

ThT fluorescence intensity (mean ± SD, n = 3) after 120 days showed significant differences among groups (one-way ANOVA, p < 0.05). HSA + Glc exhibited the highest intensity (1268 ± 40 a.u.), indicating extensive amyloid formation. In contrast, HSA + Glc + BHB showed markedly reduced fluorescence (902 ± 65 a.u., p < 0.05 vs. HSA + Glc), while control HSA (453 ± 50 a.u.) and HSA + BHB (478 ± 70 a.u.) displayed minimal ThT binding. These results demonstrate that BHB significantly attenuates glycation-induced amyloidogenesis in HSA (Fig. 5).

#### 3.3.2 Congo red binding assay

**Fig. 6:**
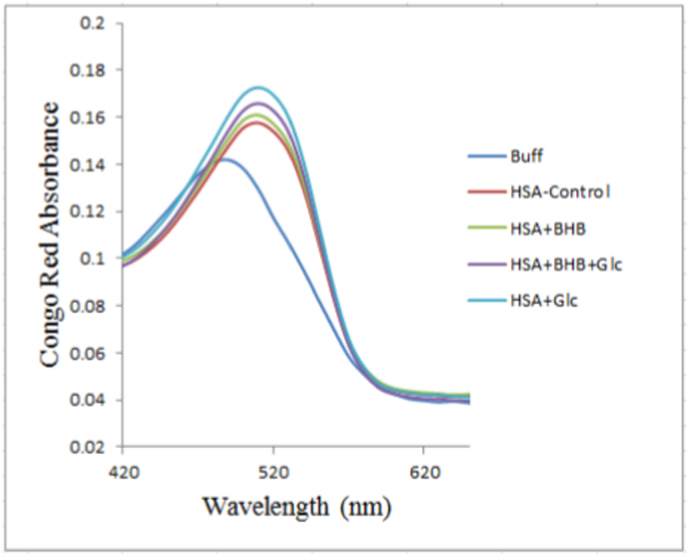
Congo red binding of HSA-control, HSA + BHB, HSA + Glc + BHB and HSA + Glc in 50 mM sodium phosphate buffer (pH 7.4), 1 mM EDTA and 0.1 mM sodium azide, incubated at 37 °C for 120 days. Data represent mean absorbance spectra from three independent experiments (n = 3). Statistical comparisons between groups were performed at the emission maximum wavelength, selected a priori as the representative spectral feature.

Congo red binding spectra of control HSA, HSA + BHB, HSA + Glc + BHB and HSA + Glc after 120 days of incubation at 37 °C are shown in Fig. 6. Relative to Congo red alone (buffer) or the control HSA sample, all modified samples displayed increased absorbance intensity accompanied by a characteristic red shift in the absorption maximum. The peak absorbance shifted from approximately 490 nm (Congo red in buffer alone) to approximately 510 nm upon binding to protein aggregates, consistent with the formation of cross-β amyloid structures. Quantitative absorbance measurements at 510 nm (mean ± SD of three independent experiments) were 0.173 ± 0.002 a.u. for HSA + Glc, 0.166 ± 0.001 a.u. for HSA + Glc + BHB (4.0% reduction, p < 0.05), 0.161 ± 0.003 a.u. for HSA + BHB, and 0.158 ± 0.001 a.u. for HSA-control. The HSA + Glc group exhibited the highest absorbance, indicating extensive cross-β amyloid structure formation, whereas the HSA + Glc + BHB group showed significantly lower absorbance, supporting BHB's anti-amyloidogenic effect.

#### 3.3.3 Morphological evaluation (AFM imaging)

**Fig. 7:**
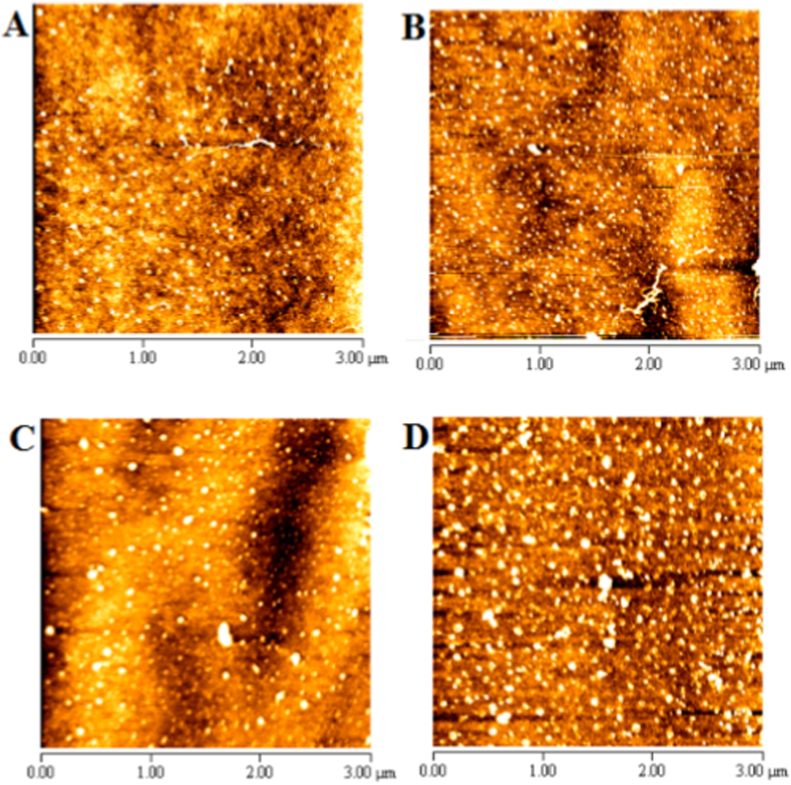
AFM images of HSA-control (A), HSA + BHB (B), HSA + Glc + BHB (C) and HSA + Glc (D) in 50 mM sodium phosphate buffer (pH 7.4), 1 mM EDTA and 0.1 mM sodium azide, incubated at 37 °C for 120 days. Images are representative of three independent experiments. Scale bar: 3 μm.

Atomic force microscopy images of control HSA, HSA + BHB, HSA + Glc + BHB, and HSA + Glc after 120 days of incubation at 37 °C are shown in Fig. 7. The control sample (panel A) displayed a relatively smooth surface with sparsely distributed small globular structures. The HSA + BHB sample (panel B) showed increased particle density and greater height heterogeneity. In the HSA + Glc + BHB sample (panel C), particle number and size were moderately increased, with loosely packed aggregates present. The HSA + Glc sample (panel D) exhibited the highest aggregation, characterized by densely packed, large particles covering the surface.

## 4 Discussion

Abnormal protein aggregation and fibrillation are central processes in the pathogenesis of numerous neurodegenerative and protein-misfolding disorders [36]. The formation of β-sheet–rich fibrillar structures not only abolishes native protein function but also leads to deposition of insoluble aggregates, disrupting cellular homeostasis and activating oxidative-stress pathways [37]. This phenomenon is well documented in Alzheimer's disease, Parkinson's disease, and systemic amyloidoses, which makes inhibition of aggregation a major biomedical objective [38]. Accumulating evidence indicates that protein glycation and subsequent formation of advanced glycation end products (AGEs) alter folding pathways and accelerate fibrillation kinetics [39]; therefore, inhibiting glycation is a promising strategy to slow amyloid-related pathology progression [40]. Here, we examined the inhibitory effects of β-hydroxybutyrate (BHB) on glycation-driven aggregation and amyloidogenesis of human serum albumin (HSA). BHB levels rise during prolonged fasting and in certain metabolic states [36]; we evaluated a physiologically relevant concentration of 21.87 mM, comparable to extended-fasting conditions.

TNBSA and AGE-specific fluorescence assays provided convergent biochemical evidence for HSA glycation under our conditions. Quantitative lysine modification analysis revealed a marked reduction in free ε-amino groups across treatments, with approximately 6 lysine residues modified per mole of HSA in the glucose-only group and ∼12 residues in the glucose + BHB group (Fig. 1). This apparently paradoxical increase in lysine modification under co-treatment conditions can be explained by the dual nature of BHB as both a glycation modulator and a reactive ketone body. BHB itself can form reversible Schiff base adducts with lysine ε-amino groups through its ketone moiety [17], in addition to glucose-derived glycation. The TNBSA assay quantifies total free lysine loss but does not distinguish between glucose-derived AGEs and BHB-lysine adducts; therefore, the co-treatment group reflects cumulative modification from both sources. Such covalent modification of lysine side chains is a canonical marker of the Maillard reaction and directly reflects glycation progression [39].

Critically, while BHB contributes to increased total lysine modification, the key distinction lies in the nature and downstream consequences of these modifications. Unlike glucose-derived AGEs, BHB-lysine adducts do not promote formation of highly fluorescent, crosslinked AGE species—as evidenced by substantially reduced AGE fluorescence in the HSA + Glc + BHB group (Fig. 2)—nor do they drive the structural destabilization and amyloidogenic aggregation observed with glucose alone (Fig. 3, Fig. 4, Fig. 5, Fig. 6, Fig. 7). This suggests that BHB's lysine modifications may represent a protective ‘competitive inhibition’ mechanism, wherein BHB occupies lysine sites that would otherwise form deleterious AGE crosslinks, thereby preventing β-sheet enrichment and fibril formation.

Complementary fluorescence spectroscopy showed pronounced emission at λex = 335 nm/λem ≈440 nm in glucose-treated samples—typical of AGE fluorophores [41]—whereas the HSA + Glc + 3BHB group exhibited substantially attenuated fluorescence (Fig. 2). The reduced AGE-specific signal in the presence of BHB suggests that, although primary lysine modification occurs, formation of downstream fluorogenic AGEs is partially suppressed. Collectively, these data confirm primary amine modification by glycation and indicate a modulatory effect of BHB on subsequent AGE-associated fluorophore formation pathways [40].

Far-UV circular dichroism analysis, as quantified by spectral deconvolution (Table 1)**,** demonstrated that glucose-mediated glycation increases total β-sheet content and decreases α-helix proportion in HSA. Representative CD spectra are shown in Fig. 3 β-Sheet enrichment is a structural hallmark associated with increased aggregation propensity, often caused by exposure of hydrophobic patches that promote intermolecular hydrophobic interactions between misfolded domains [38,42]. These interactions facilitate orderly stacking of β-sheet motifs and formation of amyloid-like fibrils [36]. Co-incubation with BHB markedly reduced the glycation-induced increase in β-sheet content and partially restored α-helical structure, consistent with attenuation of hydrophobic-interaction–driven self-assembly. This result accords with reports that small metabolites, including ketone bodies, can stabilize native folds, shield hydrophobic regions, and hinder structural transitions toward aggregation-prone conformations [43,44].

Accordingly, BHB's protective effect in the HSA + Glc + BHB group likely stems from its ability to minimize hydrophobic surface exposure and disrupt β-sheet–mediated packing, thereby mitigating glycation-driven structural destabilization and aggregation. This mechanistic interpretation aligns with previous evidence for BHB's neuroprotective and anti-amyloidogenic actions in glycation-related proteinopathies [24,45].

ANS binding measurements corroborated these structural findings. Surface hydrophobicity, assessed by ANS fluorescence at 470 nm (Fig. 4), increased 28.2% in HSA + Glc samples (459.44 ± 6 a.u., 1.28-fold, p < 0.001) compared to control (358.24 ± 5 a.u.). BHB co-treatment partially attenuated this increase to 16.9% (418.62 ± 4 a.u., 1.17-fold, p < 0.01), representing an 8.9% reduction in glycation-induced hydrophobic exposure (p < 0.05). This ∼40% attenuation of the glycation effect, though modest in absolute terms, aligns with structural stabilization observed in CD spectroscopy and reduced amyloid formation in ThT (28.9% reduction, p < 0.001) and Congo Red assays (4.0% reduction, p < 0.05). Reduced ANS signal reflects decreased solvent accessibility of hydrophobic surfaces [32], indicating that BHB masks or rearranges surface hydrophobic patches, limiting exposure and reducing aggregation propensity.

Thioflavin-T fluorescence assays quantified amyloid fibril formation after 120 days (Fig. 5). HSA + Glc samples exhibited a 6.97-fold increase in ThT fluorescence (697 ± 8 a.u. vs. control 100 ± 3 a.u., p < 0.001), indicating extensive cross-β amyloid structure formation [46]. BHB co-treatment significantly attenuated this effect to 4.96-fold (496 ± 6 a.u., p < 0.001), representing a 28.9% reduction (p < 0.001) or ∼34% attenuation of the glycation-induced amyloidogenic effect. Given that β-sheet enrichment promotes nucleation and elongation during fibrillogenesis [47], BHB's capacity to attenuate β-sheet formation (observed in CD spectroscopy) likely impedes these critical assembly phases.

Congo red binding, an orthogonal assay for cross-β structures, showed highest absorbance at 510 nm in the HSA + Glc group (0.173 ± 0.002 a.u. vs. HSA-control 0.153 ± 0.002 a.u., representing 1.13-fold increase, p < 0.01), indicating fibrillar aggregation (Fig. 6). BHB co-treatment reduced this to 0.166 ± 0.001 a.u. (p < 0.05), representing a 4.0% reduction compared to HSA + Glc. Though more modest than the ThT-measured reduction (28.9%), this statistically significant protection corroborates the structural data from CD spectroscopy and ANS binding [48]. The differential magnitude of BHB's effect between ThT and Congo red assays reflects inherent differences in dye specificity: ThT preferentially binds highly ordered cross-β amyloid fibrils with characteristic quaternary packing [46], whereas Congo red exhibits broader recognition of various β-sheet structures including less-organized aggregates and protofibrils [46,48]. Consequently, ThT fluorescence provides higher sensitivity to mature fibril content specifically disrupted by BHB, while Congo red absorbance reports on the entire spectrum of β-sheet conformations, explaining why BHB's anti-amyloidogenic effect appears more pronounced in the fibril-specific ThT assay.

Atomic force microscopy after 120 days of incubation revealed distinct morphological differences across conditions (Fig. 7). Control samples exhibited morphology similar to native HSA with sparse, small particles, indicating structural stability in the absence of glycation promoters [25]. BHB alone produced a modest increase in particle density and height heterogeneity, possibly reflecting limited surface rearrangements or BHB-related metabolic/antioxidant effects [23,49,50]. In the glucose + BHB group, AFM showed increased particle number and size relative to control but less dense packing compared to glucose-only samples, suggesting partial modulation of glycation-driven aggregation and suppression of compact fibrillar structure formation [18]. Glucose-only samples displayed the most extensive, densely packed aggregates, consistent with AGE-mediated crosslinking that reduces solubility and promotes fibrillation [18].

Overall, our results indicate that BHB acts at several stages of glycation-induced structural destabilization: it attenuates formation of fluorogenic AGEs, helps preserve α-helical secondary structure, and reduces β-sheet–driven aggregation. This multifactorial protection—demonstrated by converging biochemical, spectroscopic, and morphological evidence—supports the notion that BHB helps maintain HSA structural integrity under glycation stress. Because glycation and amyloidogenesis contribute to many age-related disorders, elucidating BHB's precise molecular mechanisms and assessing its therapeutic potential merit further investigation.

β-Hydroxybutyrate (BHB) exerts protective effects against glycation and amyloid aggregation beyond metabolic fuel function. As a signaling metabolite, BHB acts through histone deacetylase inhibition and GPR109A binding [50]. Recent studies show that ketone bodies promote amyloid-β clearance by upregulating LRP1 and P-glycoprotein [51], and reduce amyloid plaques in APP/PS1 mice via NLRP3 inflammasome inhibition [52]. While polyphenols like quercetin show up to 90% AGE inhibition in vitro [53], BHB's advantage lies in physiological accessibility—levels can be elevated through ketogenic diet, fasting, or supplementation. Unlike synthetic inhibitors with hepatotoxic risks, BHB's established safety profile supports its translational potential for glycation-induced proteinopathies in diabetes and neurodegeneration.

Several limitations of this study warrant acknowledgment. First, while our multi-technique approach (AFM, ThT, Congo Red) demonstrates BHB's protective effects against mature fibril formation, we cannot definitively exclude the presence of pre-fibrillar oligomeric intermediates—often the most cytotoxic species in amyloid pathways [54]. Future studies incorporating transmission electron microscopy (TEM), size-exclusion chromatography (SEC), dynamic light scattering (DLS), and A11 antibody immunoblotting would enable higher-resolution characterization of aggregate size distributions and detection of soluble oligomers [55]. Second, although our data demonstrate reduced fibril formation in the presence of BHB, we did not assess cytotoxicity endpoints; therefore, the biological significance of the observed structural protection remains to be validated in neuronal or endothelial cell-based models to clarify whether BHB-treated HSA retains toxic oligomeric conformers [56]. Third, the physiological relevance of 21.87 mM BHB—while achievable during prolonged fasting or ketogenic states—requires verification in vivo, particularly regarding tissue-specific accumulation and long-term safety [23]. Addressing these gaps will be essential to establish BHB's therapeutic potential in glycation-related disorders.

## 5 Conclusion

This study demonstrates that prolonged glycation of human serum albumin (HSA) by glucose induces profound structural destabilization, characterized by α-helix loss, β-sheet enrichment, increased surface hydrophobicity, and extensive amyloid-like aggregation. Across multiple complementary techniques—including AGE quantification, circular dichroism spectroscopy, ANS binding, Thioflavin T fluorescence, Congo red absorption, and atomic force microscopy—β-hydroxybutyrate (BHB) consistently attenuated these glycation-driven changes. Co-incubation with BHB preserved secondary structure, limited β-sheet-rich conformer formation, reduced hydrophobic exposure, and mitigated fibril deposition, yielding less compact aggregate morphologies.

The convergence of biochemical, spectroscopic, and morphological evidence supports a multifaceted protective role of BHB, likely involving structural stabilization, modulation of aggregation pathways, and reduction of downstream oxidative stress. These findings position BHB as a promising biochemical modulator against glycation-induced protein aggregation, with potential relevance to amyloid-associated neurodegenerative disorders such as Alzheimer's disease. Future studies should dissect the precise molecular mechanisms underlying BHB's actions and evaluate its translational potential in physiological and pathological contexts.

## Funding

This research was financially supported by the 10.13039/501100017252University of Zabol under grant number UOZ-GR-5866. The grant was used to cover the costs of materials and laboratory analyses. The funding source had no involvement in the study design, data collection and analysis, interpretation of results, manuscript writing, or in the decision to submit the manuscript for publication.

## CRediT authorship contribution statement

**Hojjat Mohammadnia:** Investigation. **Mousa Bohlooli:** Conceptualization, Data curation, Formal analysis, Investigation, Methodology, Project administration, Supervision, Writing – original draft, Writing – review & editing. **Mansour Ghaffari-Moghaddam:** Data curation, Formal analysis. **Mostafa Khajeh:** Data curation, Formal analysis. **Fereshteh Taghavi:** Conceptualization, Writing – review & editing.

## Declaration of competing interest

There are no known competing financial interests or personal relationships that could have influenced the findings in this paper.

## Data Availability

All data generated or analyzed during this study are included in this published article.
